# Immune biomarkers of response and resistance to ipilimumab and nivolumab in metastatic castration-resistant prostate cancer

**DOI:** 10.1126/sciadv.aee8730

**Published:** 2026-10-14

**Authors:** Apostolia M. Tsimberidou, Christopher R. Cabanski, Alexandra Drakaki, Danny N. Khalil, Bilal A. Siddiqui, Sreyashi Basu, Kwame Okrah, Diane M. Da Silva, Shikha Gautam, Farah A. Alayli, Mike Schachter, Justin Fairchild, Wai Chin Foo, Silvia Boffo, Jamie Arnott, Ute Dugan, Sumit K. Subudhi, Padmanee Sharma

**Affiliations:** ^1^ Department of Investigational Cancer Therapeutics, The University of Texas MD Anderson Cancer Center, Houston, TX, USA.; ^2^ Parker Institute for Cancer Immunotherapy, San Francisco, CA, USA.; ^3^ University of California, Los Angeles Jonsson Comprehensive Cancer Center, Los Angeles, CA, USA.; ^4^ Department of Medicine, Memorial Sloan Kettering Cancer Center, New York, NY, USA.; ^5^ Department of Genitourinary Medical Oncology, The University of Texas MD Anderson Cancer Center, Houston, TX, USA.; ^6^ James P. Allison Institute, The University of Texas MD Anderson Cancer Center, Houston, TX, USA.; ^7^ Immunotherapy Platform, The University of Texas MD Anderson Cancer Center, Houston, TX, USA.; ^8^ Department of Pathology, The University of Texas MD Anderson Cancer Center, Houston, TX, USA.; ^9^ Bristol Myers Squibb, New York, NY, USA.; ^10^ Department of Immunology, The University of Texas MD Anderson Cancer Center, Houston, TX, USA.

## Abstract

Immune checkpoint therapies have shown limited efficacy in metastatic castration-resistant prostate cancer (mCRPC). The AMADEUS prostate expansion cohort (NCT03651271) evaluated the efficacy, safety, and biomarkers of escalating ipilimumab doses combined with nivolumab. Patients received ipilimumab 1 milligram per kilogram (mg/kg) + nivolumab 360 mg (I1+N360), ipilimumab 3 mg/kg + nivolumab 1 mg/kg (I3+N1), or ipilimumab 5 mg/kg + nivolumab 1 mg/kg (I5+N1). Coprimary endpoints were 6-month disease control rate (DCR) and changes in tumoral CD8 percentage. Thirty-two patients were enrolled. DCRs were 33% (4 of 12) for I1+N360, 20% (1 of 5) for I3+N1, and 20% (3 of 15) for I5+N1. Across cohorts, tumoral CD8 percentage increased by a median of 5.0%. Although higher ipilimumab doses were tolerable, I1+N360 achieved similar disease control and immune activation with a more favorable safety profile. Early disease progression was associated with increased expression of a TREM2-associated myeloid gene signature, which was also significantly enriched in two independent prostate cancer datasets.

## INTRODUCTION

Prostate cancer is the most common non–skin cancer among American men, accounting for 31% of new cancer cases (*1*). While early-stage prostate cancer typically has a favorable prognosis, in approximately one-third of patients, it progresses to metastatic disease, which is incurable. Metastatic prostate cancer inevitably progresses to metastatic castration-resistant prostate cancer (mCRPC), the lethal form of the disease, with a median life expectancy of approximately 2 years (*2*).

Immune checkpoint therapies (ICTs) targeting pathways such as CTLA-4, PD-(L)1, and LAG3 have revolutionized cancer therapy by extending survival in various malignancies (*3*–*5*). However, their efficacy in mCRPC has been limited, with only modest responses observed in clinical trials targeting PD-1, PD-L1, and CTLA-4 (*6*–*12*). The limited success of ICTs in mCRPC is attributed mostly to its “immunologically cold” nature, characterized by low tumor mutational burden (TMB), sparse T cell infiltration within the tumor microenvironment (TME), diminished natural killer (NK) cell activity, and the presence of immunosuppressive myeloid cells (*13*–*16*). Combining ipilimumab (anti–CTLA-4) with nivolumab (anti–PD-1) has the potential to convert tumors from “cold” to “hot” by increasing intratumoral CD8^+^ T cells in the prostate TME (*17*). In addition, studies have shown that increased tumoral CD8^+^ T cells posttreatment were associated with improved clinical outcomes (*18*, *19*).

Several clinical trials have demonstrated durable tumor responses in patients with mCRPC treated with nivolumab plus ipilimumab (*20*–*22*), including initial encouraging activity using ipilimumab 1 mg/kg within the AMADEUS study (*19*). Building on these results and given the potential for higher ipilimumab doses to enhance T cell priming and overcome resistance, we designed the AMADEUS prostate expansion cohort to evaluate the safety and efficacy of increasing ipilimumab doses (3 and 5 mg/kg) in combination with nivolumab. This study also aimed to provide deeper insights into potential mechanisms of response and resistance to combination immunotherapy in mCRPC.

## RESULTS

### Trial design and patient characteristics

From 16 January 2019 through 4 February 2020, 12 patients with mCRPC were enrolled into the initial solid tumor cohort of the AMADEUS trial and received combination nivolumab at 360 mg and up to four doses of ipilimumab at 1 mg/kg (I1+N360; Fig. 1) (*19*). On the basis of preliminary clinical activity observed in these patients, we initiated an expansion cohort to investigate higher doses of ipilimumab in patients with mCRPC. From 3 March 2021 through 29 November 2021, 10 patients with mCRPC were randomized 1:1 to receive nivolumab at 1 mg/kg in combination with up to 2 doses of ipilimumab at 3 mg/kg (I3+N1) or 5 mg/kg (I5+N1). After a safety pause, 10 additional patients were enrolled to the I5+N1 cohort, with enrollment concluding on 17 October 2022.

**Fig. 1.**
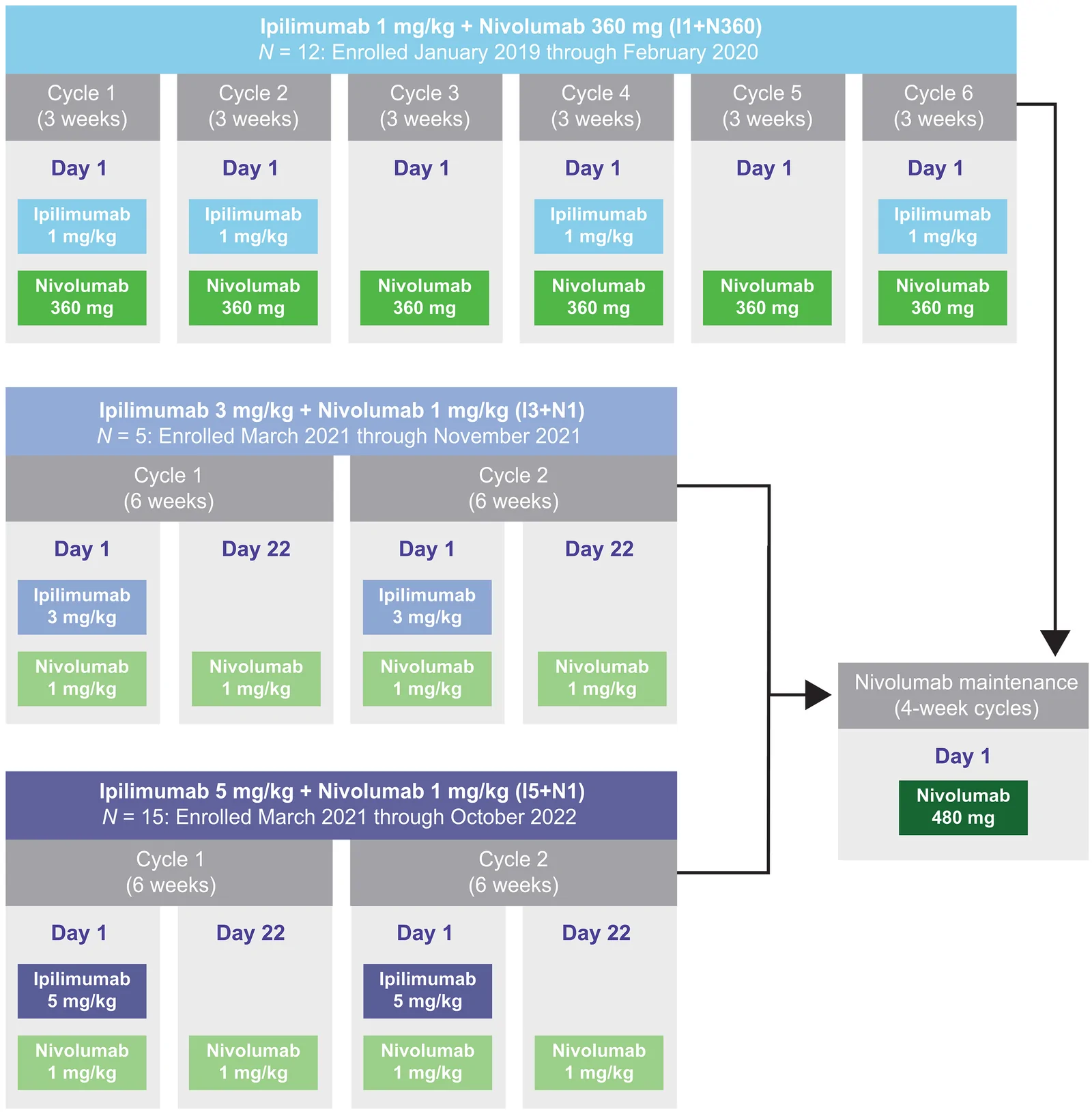
Dosing schedule. Patients with mCRPC received nivolumab and ipilimumab combination. After completing six 3-week cycles in the I1+N360 cohort or two 6-week cycles in the I3+N1 and I5+N1 cohorts, patients continued to receive maintenance nivolumab until disease progression or intolerable toxicity. Tumor biopsies were mandatory at screening. If medically feasible, on-treatment biopsies were collected during cycles 2 and 6 in I1+N360, and within 3 days of cycle 1 day 22 and cycle 2 day 22 in I3+N1 and I5+N1.

The primary endpoints were the 6-month disease control rate (DCR) and change from baseline in the tumoral CD8 cell percentage by immunohistochemistry (IHC). Secondary endpoints included the objective response rate (ORR), progression-free survival (PFS), overall survival (OS), and the incidence and severity of adverse events (AEs). Efficacy and safety were assessed for all 32 enrolled patients. As of 16 October 2023 (cutoff date for data analysis), all patients had discontinued treatment and follow-up.

Patient demographic and baseline characteristics were well balanced between the I1+N360 and I5+N1 cohorts while patients assigned to I3+N1 were generally older and had higher baseline PSA levels and tumoral CD8 percentage (Table 1). Patients were heavily pretreated, having received a median of five prior lines of systemic treatment in I1+N360 and I5+N1 and nine lines in I3+N1 (table S1). Thirty-one (96.9%) patients received a second-generation androgen receptor pathway inhibitor (ARPI). Across all cohorts, four (12.5%) patients had received prior anti–PD-(L)1, although the anti–PD-(L)1 treatment was not the most recent line of therapy (adhering to eligibility criteria).

**Table 1.** Baseline demographic and clinical characteristics of patients with mCRPC across ipilimumab dose-escalation cohorts in the AMADEUS trial. Data are summarized for all enrolled patients (*N* = 32) and stratified by treatment arm: ipilimumab 1 mg/kg + nivolumab 360 mg (I1+N360, *N* = 12), ipilimumab 3 mg/kg + nivolumab 1 mg/kg (I3+N1, *N* = 5), and ipilimumab 5 mg/kg + nivolumab 1 mg/kg (I5+N1, *N* = 15). Continuous variables are reported as median (range), and categorical variables are reported as absolute count (*n*) and percentage (%). Abbreviations: ARPI, androgen receptor pathway inhibitor; CTLA-4, cytotoxic T-lymphocyte–associated protein 4; ECOG, Eastern Cooperative Oncology Group; mCRPC, metastatic castration-resistant prostate cancer; PD-(L)1, programmed cell death (ligand) 1; PSA, prostate-specific antigen; ULN, upper limit of normal.

	I1+N360 (*N* = 12)	I3+N1 (*N* = 5)	I5+N1 (*N* = 15)	Total (*N* = 32)
Age (years), median (range)	69 (50–80)	74 (63–84)	68 (53–79)	71.5 (50–84)
Race, *n* (%)				
Black or African American	3 (25)	1 (20)	1 (7)	5 (16)
White	9 (75)	3 (60)	14 (93)	26 (81)
Other	0	1 (20)	0	1 (3)
Hispanic or Latino, *n* (%)	0	0	0	0
ECOG performance status, *n* (%)				
0	5 (42)	0	4 (27)	9 (28)
1	7 (58)	5 (100)	11 (73)	23 (72)
Prior lines of systemic cancer therapy, median (range)	5 (2–8)	9 (3–10)	5 (2–8)	5 (2–10)
Prior second-generation ARPI, *n* (%)	11 (92)	5 (100)	15 (100)	31 (97)
Prior anti–PD-(L)-1, n (%)	1 (8)	1 (20)	2 (13)	4 (13)
Prior anti–CTLA-4, n (%)	1 (8)	0	0	1 (3)
Baseline tumor biopsy site, *n* (%)				
Lymph node	5 (42)	4 (80)	12 (80)	21 (66)
Liver	1 (8)	0	1 (7)	2 (6)
Lung	1 (8)	0	1 (7)	2 (6)
Soft tissue (e.g., abdominal wall and muscle)	2 (16)	1 (20)	0	3 (9)
Other	3 (25)	0	1 (7)	4 (13)
Tumoral CD8 cells (%), median (range)	3.0 (0–13.0)	4.5 (0.1–10.1)	3.3 (0.1–14.1)	3.4 (0–14.1)
PSA (ng/ml), median (range)	70.0 (0–863.7)	196.9 (10.8–4271.0)	31.2 (0.2–2001.0)	58.3 (0–4271.0)
Alkaline phosphatase, *n* (%)				
<1.5 × ULN	10 (83)	4 (80)	11 (73)	25 (78)
≥1.5 × ULN	2 (17)	0	4 (27)	6 (19)
Not reported	0	1 (20)	0	1 (3)
Hemoglobin, *n* (%)				
<11 g/dl	4 (33)	1 (20)	5 (33)	10 (31)
≥11 g/dl	8 (67)	4 (80)	10 (67)	22 (69)
Lactate dehydrogenase, *n* (%)				
<200 U/liter	2 (17)	1 (20)	5 (33)	8 (25)
≥200 U/liter	10 (83)	2 (40)	7 (47)	19 (59)
Not reported	0	2 (40)	3 (20)	5 (16)

All patients received at least one dose of ipilimumab and nivolumab. For ipilimumab, the cumulative exposure increased in alignment with the planned dosing regimens across study cohorts. Specifically, the median cumulative doses were 228, 480, and 690 mg for I1+N360, I3+N1, and I5+N1, respectively (table S2). Although 83.3% of patients in I1+N360 received at least two doses of ipilimumab, only 25.0% completed all four planned doses. In contrast, 60.0% of patients in I3+N1 and 53.3% in I5+N1 received both planned doses. For nivolumab, despite patients in all cohorts receiving a median of three doses, cumulative exposure was approximately three- to fourfold higher in I1+N360 due to the fixed-dose strategy, independent of patient weight.

### Clinical activity

Clinical activity was observed in this heavily pretreated patient population. The ORR across all three cohorts was 9.4% [95% confidence interval (CI): 2.0 to 25.0], with two partial responses (PRs) observed in I1+N360 and one in I5+N1 (Fig. 2A and Table 2). The 6-month DCR was 25.0% (95% CI: 11.5 to 43.4), median radiographic PFS was 5.4 months (95% CI: 4.3 to 9.4), and median OS was 27.9 months (95% CI: 7.9 to not estimable). This study was not powered for direct comparisons between treatment cohorts, and all such comparisons should be interpretated as exploratory. Nonetheless, the rates of response, PFS, and OS were numerically comparable.

**Fig. 2.**
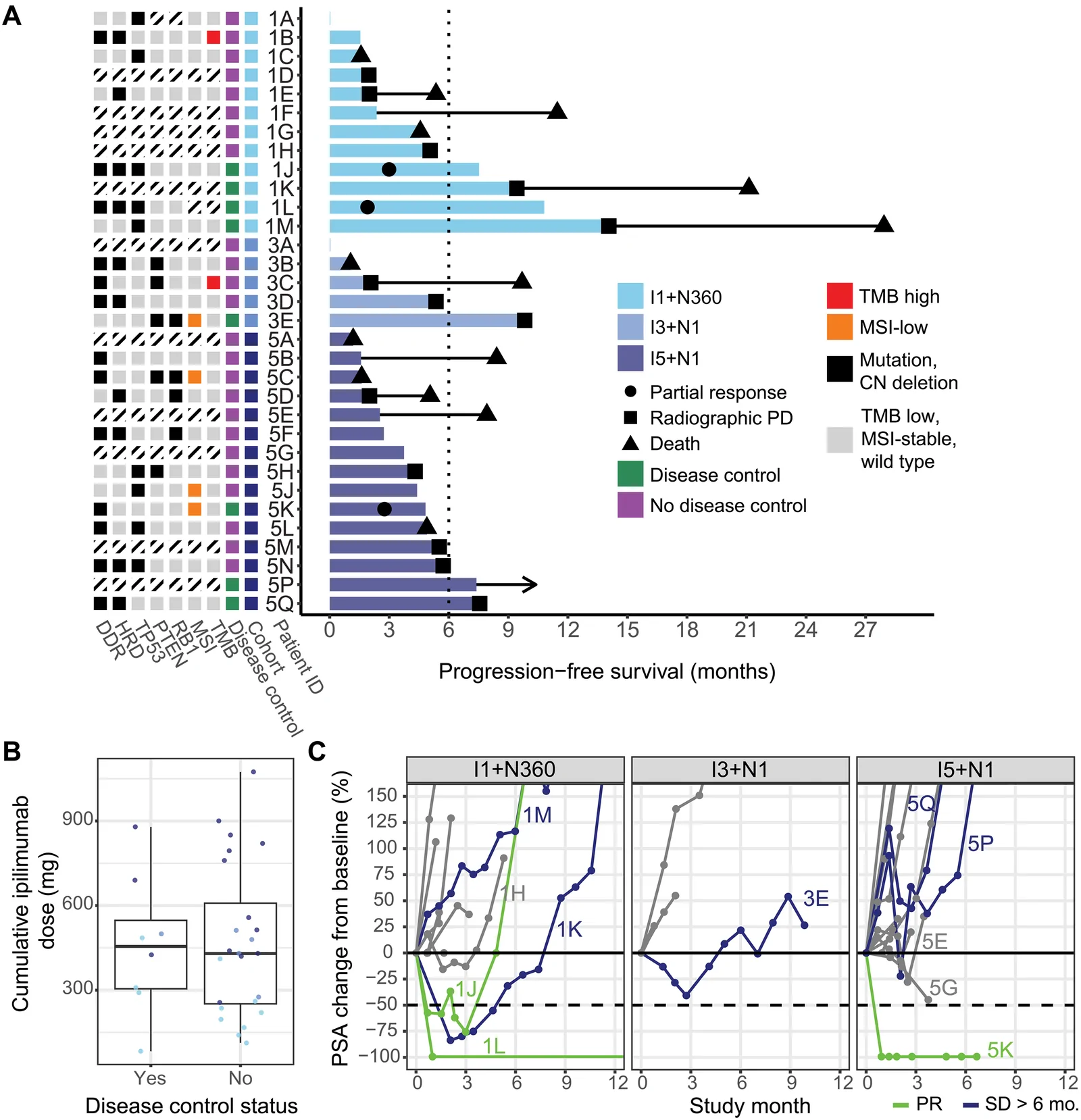
Clinical activity. (**A**) Left: TMB, MSI status, copy number deletion of *PTEN* and *RB1*, and mutations in *TP53*, HRD genes, and DDR genes via WES. Patients without WES data are denoted by hash lines. Right: PFS time for each of the 32 enrolled patients is indicated by the length of the colored bar. Circles, squares, and triangles denote the time of first PR, radiographic disease progression (PD), and death, respectively. One patient (ID: 5P in I5+N1) had ongoing SD at the time of study closure. Two patients were censored on day 1 due to withdrawal of consent (ID: 1A in I1+N360) and clinical progression (ID: 3A in I3+N1). Both patients received only one dose of ipilimumab and nivolumab and did not undergo postbaseline radiographic assessment. (**B**) Cumulative ipilimumab dose received, stratified by disease control status and data points colored by cohort. Box plots show the median and quartiles, and whiskers extend to the largest value no further than 1.5 times the interquartile range. (**C**) Percent change from baseline in PSA levels for patients with at least one on-treatment PSA measurement (*n* = 28). Green lines designate patients who achieved a PR and blue lines designate patients who achieved SD for at least 6 months. Select lines are annotated with the corresponding patient ID. Patient 1L maintained a >99% decrease in PSA for more than 19 months.

**Table 2.** Clinical activity, PSA dynamics, and tumoral CD8 T cell changes across ipilimumab dose cohorts in the AMADEUS trial. Summary of clinical outcomes, prostate-specific antigen (PSA) kinetics, and paired biopsy biomarker changes for all enrolled patients (*N* = 32) and stratified by treatment arm: I1+N360 (*N* = 12), I3+N1 (*N* = 5), and I5+N1 (*N* = 15). Time-to-event endpoints and response rates are presented with 95% confidence intervals (CIs). Continuous percentage changes and CD8 metrics are reported as median [interquartile range (IQR)]. Categorical variables are reported as absolute count (*n*) and percentage (%). Abbreviations: CI, confidence interval; CR, complete response; IQR, interquartile range; NE, not estimable; NR, not reached; PR, partial response; PSA, prostate-specific antigen; SD, stable disease.

	I1+N360 (*N* = 12)	I3+N1 (*N* = 5)	I5+N1 (*N* = 15)	Total (*N* = 32)
Best response, *n* (%)				
Complete response (CR)	0	0	0	0
Partial response (PR)	2 (16.7)	0	1 (6.7)	3 (9.4)
Stable disease (SD)	6 (50.0)	2 (40.0)	11 (73.3)	19 (59.3)
Progressive disease	2 (16.7)	1 (20.0)	1 (6.7)	4 (12.5)
No available posttreatment tumor assessment*	2 (16.7)	2 (40.0)	2 (13.3)	6 (18.8)
Objective response rate (95% CI), %	16.7 (2.8–48.4)	0 (0.0–52.2)	6.7 (0.2–31.9)	9.4 (2.0–25.0)
Six-month disease control rate (95% CI), %	33.3 (9.9–65.1)	20.0 (0.5–71.6)	20.0 (4.3–48.1)	25.0 (11.5–43.4)
Progression-free survival, months				
Median (95% CI)	5.1 (1.6–NE)	3.7 (1.1–NE)	5.5 (2.0–NE)	5.4 (4.3–9.4)
Overall survival, months				
Median (95% CI)	27.9 (4.6–NE)	NR (1.1–NE)	NR (4.9–NE)	27.9 (7.9–NE)
PSA (ng/ml)				
Number with on-treatment PSA value (%)	11 (91.7)	4 (80.0)	13 (86.7)	28 (87.5)
Best percent change from baseline, median (IQR), %	0.0 (−45.7 to 27.6)	32.6 (9.3–50.4)	13.4 (−22.1 to 48.7)	7.0 (−23.5 to 44.3)
Reduction ≥ 50%, *n* (%)	3 (25.0)	0	1 (6.7)	4 (12.5)
Tumoral CD8 percentage				
Patients with on-treatment biopsy, *n* (%)	6 (50.0)	3 (60.0)	8 (53.3)	17 (53.1)
Baseline tumoral CD8, median (IQR), %	1.5 (1.0–3.5)	5.5 (5.0–7.8)	2.6 (2.1–4.9)	2.8 (1.6–5.0)
On-treatment tumoral CD8, median (IQR), %	12.0 (4.3–19.8)	3.3 (3.2–13.2)	11.8 (5.9–14.8)	8.8 (3.1–16.0)
Change from baseline, median (IQR)	9.5 (2.5–18.8)	−1.2 (−1.8 to 5.9)	5.0 (0.1–11.7)	5.0 (0.0–12.1)

* Reasons for patients not having postbaseline tumor assessments include death (*n* = 3), withdrawal of consent (*n* = 2), and clinical deterioration (*n* = 1).

When pooling across cohorts, baseline characteristics were generally similar between patients who achieved disease control (*n* = 8) and those who did not (*n* = 24) (table S3 and data file S1). Patients with disease control had significantly lower lactate dehydrogenase levels (*P* = 0.027) and significantly higher hemoglobin levels (*P* = 0.035) at baseline compared to patients who did not achieve disease control, consistent with prior subgroup analysis of a phase 3 clinical trial of ipilimumab in mCRPC (*9*). Although not reaching statistical significance, patients with disease control were generally older and white, with an Eastern Cooperative Oncology Group (ECOG) performance status of 0, lower baseline serum levels of prostate-specific antigen (PSA) and alkaline phosphatase, and fewer lines of prior systemic therapy. Baseline tumoral CD8 levels and prior exposure to anti–PD-(L)1 therapy were not associated with disease control. In addition, the cumulative dose of ipilimumab received was not associated with disease control (Fig. 2B). For patients with available whole exome sequencing (WES) at baseline (*n* = 20), disease control was not associated with TMB, microsatellite instability (MSI) status, copy number loss of *PTEN* or *RB1*, or mutations in *TP53*, homologous recombination deficiency (HRD) genes, or DNA damage response (DDR) genes.

Serum PSA levels were measured longitudinally for 28 patients (87.5%). Most patients experienced PSA increases within 3 months of treatment initiation. However, three patients in I1+N360 [two PRs and one stable disease (SD) exceeding 6 months] and one patient in I5+N1 (PR) experienced PSA reductions exceeding 50% (Fig. 2C).

### Safety

The frequency and severity of treatment-emergent adverse events (TEAEs) were similar across cohorts and consistent with the established safety profile of combination ipilimumab and nivolumab. Notably, no increases in the overall AE rate or severity were observed in cohorts that administered higher doses of ipilimumab. All patients (100%) experienced at least one AE. Fatigue (40.6%) and rash (34.4%) were the most frequently observed AEs across the cohorts (table S4). Grade 3 or 4 AEs occurred in 75.0% (*n* = 9), 60.0% (*n* = 3), and 66.7% (*n* = 10) of patients in each respective cohort. Two grade 5 AEs were observed (cardio-respiratory arrest in I1+N360 and myocardial infarction in I3+N1), both deemed unrelated to study treatment by the investigator. Across all cohorts, there was no association between grade 3 or 4 treatment-related AEs and disease control (two-sided Fisher’s exact test, *P* = 1.00).

Nine (28.1%) patients discontinued treatment owing to an AE, including four (33.3%) in I1+N360 and five (33.3%) in I5+N1 (table S5). Seven of the AEs that resulted in treatment discontinuation were treatment related (two in I1+N360 and five in I5+N1). Three of the discontinuations, all in I5+N1, were due to immune-mediated colitis.

### Candidate pharmacodynamic markers

Seventeen (53.1%) of 32 patients had an on-treatment tumor biopsy. Biopsies were not obtained for the remaining 15 patients due to medical ineligibility, patient refusal, or early study discontinuation. On-treatment biopsies were obtained from the same anatomical site as baseline. Consistent with our hypothesis, tumoral CD8 percentage increased after ipilimumab and nivolumab treatment. Pretreatment tumors were “immunologically cold,” with a median CD8 percentage of 2.8% [interquartile range (IQR): 1.6 to 5.0]. After treatment initiation, the median change in tumoral CD8 percentage from baseline was +5.0% (IQR: 0.0 to 12.1). Tumors treated with higher doses of ipilimumab did not have meaningfully higher increases in CD8 frequencies (Table 2 and Fig. 3A). In addition, changes in tumoral CD8 percentage were not associated with response.

**Fig. 3.**
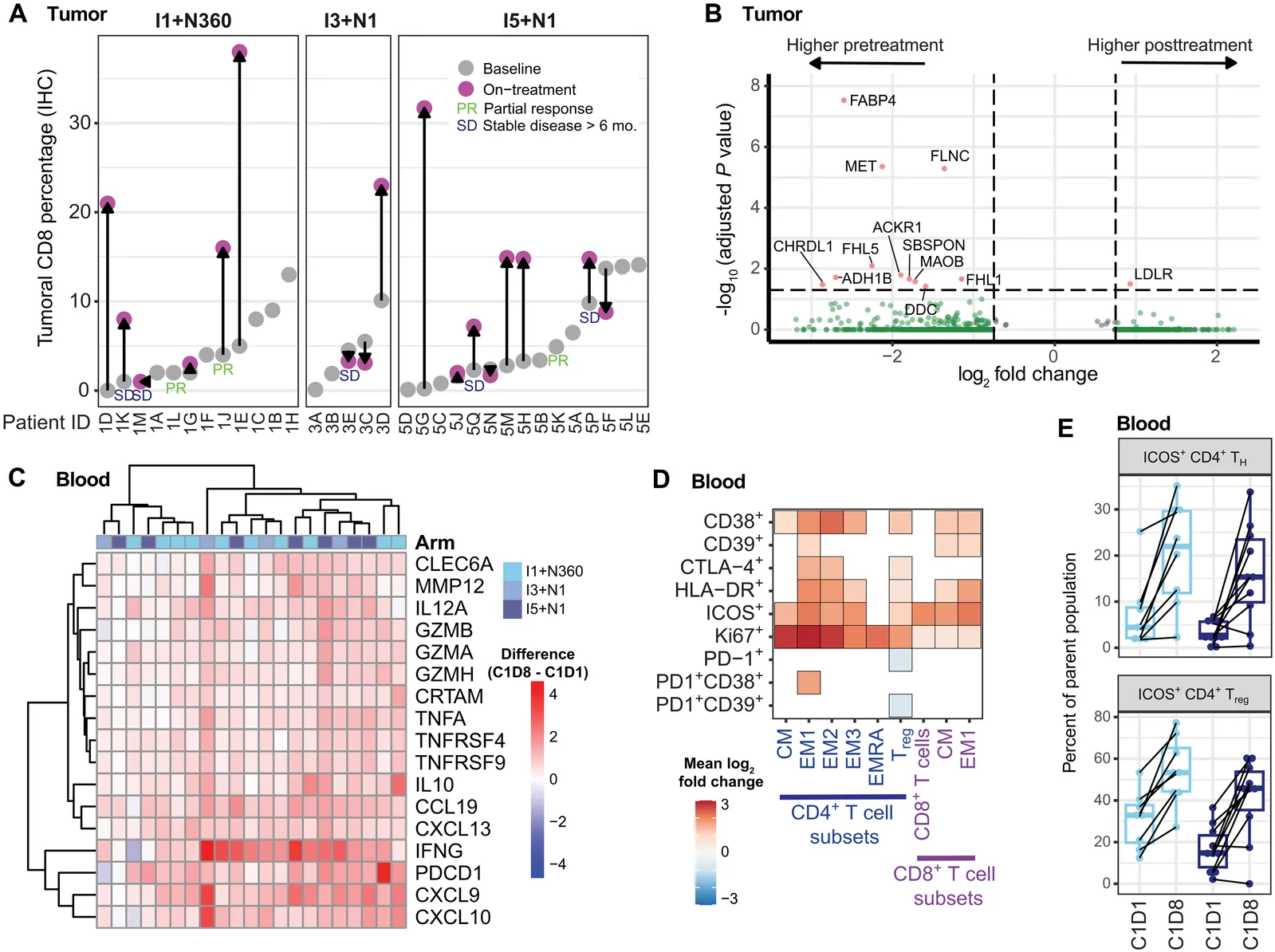
Changes in tumor- and blood-based candidate biomarkers in response to ipilimumab and nivolumab. (**A**) Changes in tumoral CD8 percentage, measured using IHC. Gray and magenta circles indicate pretreatment and on-treatment CD8 percentages, respectively. Patients who achieved disease control are designated by a PR (partial response) or SD (stable disease ≥ 6 months) symbol. (**B**) Volcano plot of differential gene expression between paired pre- and posttreatment tumor biopsies (*n* = 6). The horizontal line indicates the significance threshold corresponding to a Benjamini-Hochberg adjusted *P* value of 0.05. (**C**) Heatmap showing the absolute change in expression levels at cycle 1 day 8 (C1D8) from pretreatment [cycle 1 day 1 (C1D1)] for serum proteins that were significantly different when pooling across all treatment cohorts (Bonferroni-adjusted two-sided *t* test *P* value <0.05 and mean difference > 0.5; *n* = 21). (**D**) Mean log_2_ fold change percent-of-parent abundance for CD4^+^ and CD8^+^ T cell populations that were significantly different when pooling across all treatment cohorts (Bonferroni-adjusted two-sided *t* test *P* value <0.05 and log2 fold change >0.5; *n* = 22). (**E**) Boxplots of ICOS^+^ cells as a percentage of CD4^+^ T helper (FoxP3^−^) and CD4^+^ T regulatory (FoxP3^+^ CD25^+^) cells at C1D1 and C1D8. Box plots show the median and quartiles, and whiskers extend to the largest value no further than 1.5 times the interquartile range. CM, central memory; EM, effector memory; EMRA, terminally differentiated effector memory; T_H_, T helper cell; T_reg_, regulatory T cell.

Bulk RNA sequencing (RNA-seq) analyses of tumor samples were performed, with the three treatment cohorts pooled due to the limited number of matched pre- and on-treatment samples (*n* = 1, 1, and 4, respectively, including 2 who achieved disease control). Twelve genes were differentially expressed between the pre- and on-treatment tumors (Fig. 3B), including *FABP4*, which is implicated in immune metabolism. Its down-regulation posttreatment likely reflects metabolic reprogramming and immune cell function changes within the TME (*23*). Gene set enrichment analysis (GSEA) revealed significant down-regulation of immune-related pathways, cellular metabolism, and tumor progression mechanisms following ipilimumab and nivolumab treatment (fig. S1).

Serum proteins were measured pretreatment [cycle 1 day 1 (C1D1)] and 7 days after the first dose of ipilimumab and nivolumab [cycle 1 day 8 (C1D8)]. Pooling across cohorts (*n* = 21) revealed 17 proteins that were significantly up-regulated on-treatment, aligning with the anticipated pharmacodynamic effects of ipilimumab and nivolumab (Fig. 3C). Notably, these included cytokines and chemokines such as CCL19, CXCL9, CXCL10, and CXCL13, which are known to modulate T cell activation and recruitment to the TME (*24*). In addition, increases in immune regulatory cytokines including IFNG, IL12A, and TNFA, alongside the proteases GZMA, GZMB, and GZMH, suggest activation of both pro-inflammatory responses and cytolytic activity (*25*, *26*). The up-regulation of PDCD1 may represent a compensatory feedback response to CTLA-4 blockade, as previously described (*17*). No significant down-regulated proteins were identified.

Broad immune profiling of peripheral blood mononuclear cells was performed using mass cytometry. Pooling data across treatment cohorts (*n* = 22) revealed significant alterations in the expression of various T cell populations from C1D1 to C1D8 (Fig. 3D). Proliferative markers such as Ki67 were up-regulated in multiple T cell subsets, including CD4^+^ T helper cells and regulatory T cells (T_reg_ cells), reflecting a robust cell-mediated immune response. Increases in activation and costimulatory markers such as ICOS across CD8^+^ and CD4^+^ T cell compartments, particularly in central memory (CM) and effector memory (EM) phases, were prominent.

Next, we investigated differences in the magnitude of pharmacodynamic effects of serum proteins and peripheral blood T cell populations between the I1+N360 and I5+N1 cohorts. Eight proteins were differentially expressed between cohorts (fig. S2A). While some differences in dynamics may have been driven by baseline imbalances, several other immune-related proteins, including CSF1 and TNFRSF12A, increased on-treatment in I5+N1 only, suggesting that lower doses of ipilimumab may not sufficiently trigger their elevation. Fourteen differential immune cell populations were identified, but many showed minimal changes or were driven by baseline imbalances. However, four populations exhibited statistically significant and biologically plausible changes (fig. S2B). Patients in I1+N360, who received a higher dose of nivolumab, showed increases in PD-1^+^CD38^+^ CD4^+^ T helper EM (EM3; CD45RA^−^CD27^−^CCR7^−^) and terminally differentiated EM (EMRA; CD45RA^+^CD27^−^CCR7^−^) cells, consistent with enhanced T cell activation. Conversely, patients in I5+N1, who received a higher dose of ipilimumab, demonstrated increases in CD8^+^ EM (CCR7^−^) cells and Ki67^+^ CD141^+^ myeloid dendritic cells, reflecting the role of ipilimumab in promoting T cell and dendritic cell responses. Notably, while ICOS^+^ CD4^+^ T cells increased across all cohorts, no dose-dependent differences were observed (Fig. 3E).

### Candidate pretreatment biomarkers of disease control in tumor tissue

To identify pretreatment predictive biomarkers, we performed an unbiased RNA-seq analysis on tumor biopsies from patients with clearly distinct clinical outcomes. This bookend analysis focused on patients with documented evidence of radiographic or clinical progression within 4 months (*n* = 5) and those achieving a PR or SD for at least 6 months (*n* = 4). CIBERSORT analysis did not demonstrate a statistically significant difference in the relative abundance of immune cell populations across samples, likely due to the limitations of bulk transcriptional data in resolving the identities of specific cellular subsets (fig. S3). Differential gene expression analysis revealed 122 genes that were significantly altered between these two groups (Fig. 4A and data file S2). These results identified distinct gene expression patterns, with immune-modulatory and stress response genes such as *MMP7*, *SERPINA3*, and *CHI3L1* significantly enriched in patients with early progression. This suggests that early progressors may exhibit a dysregulated immune environment with elevated stress signaling that is unable to be reversed by ipilimumab and nivolumab. GSEA revealed pathways positively enriched in patients with disease control, including those related to complement and coagulation cascades, cytokine-cytokine receptor interaction, inflammatory response, and interferon gamma response, indicating a more inflamed and immune-active TME at baseline in these tumors (fig. S4). Exclusion of the single non–lymph node sample from this bookend analysis yielded similar overall transcriptomic patterns.

**Fig. 4.**
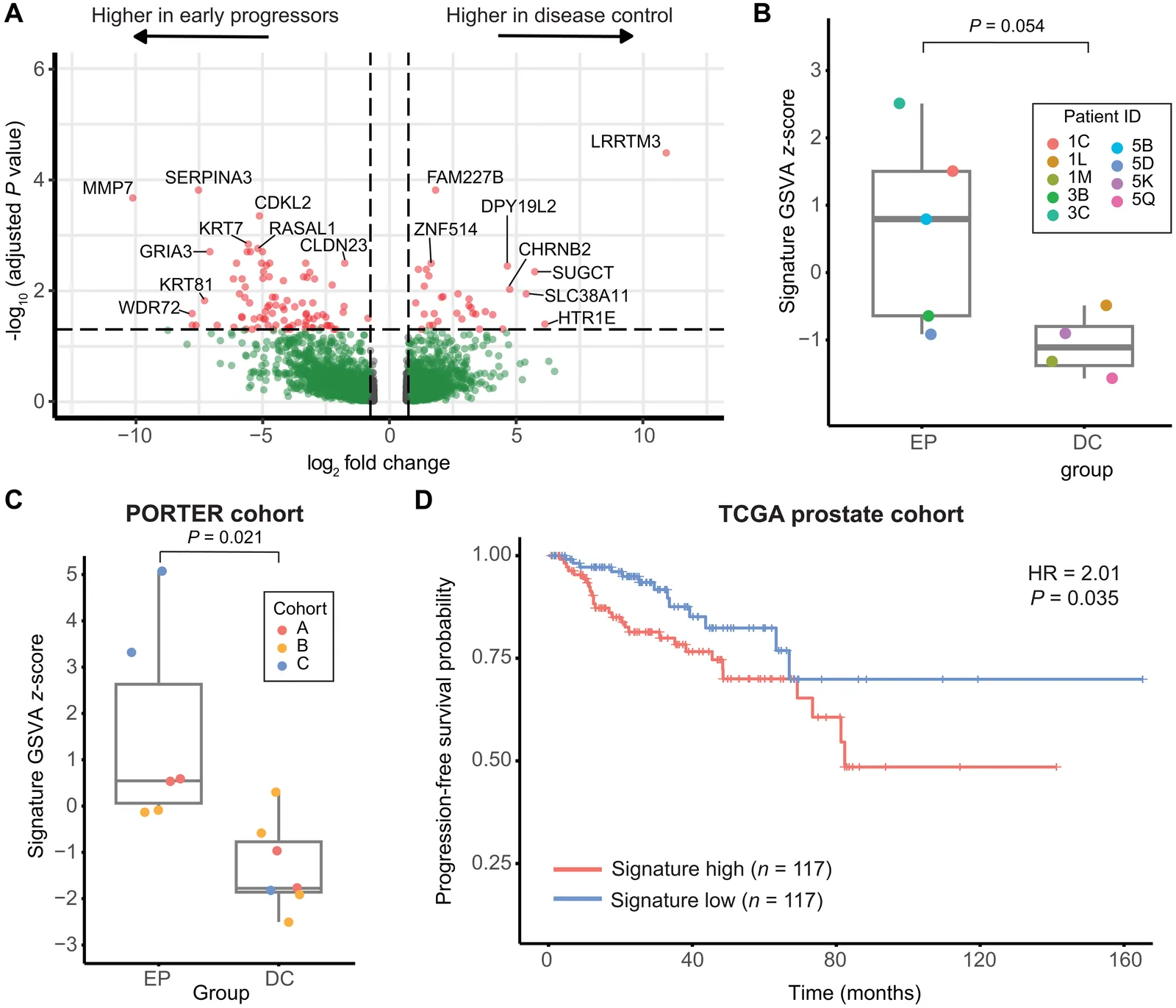
Baseline tumor biomarkers and the TREM2-associated myeloid signature correlate with clinical outcome in metastatic castration-resistant prostate cancer. (**A**) Volcano plot of differential gene expression from pretreatment tumor RNA-seq between patients achieving disease control (DC) for at least 6 months (*n* = 4) and those with clinical or radiographic progression within 4 months [early progressors (EP); *n* = 5]. The horizontal line indicates the significance threshold corresponding to a Benjamini-Hochberg adjusted *P* value of 0.05. (**B**) TREM2-associated gene signature for patients in the AMADEUS trial who achieved DC for at least 6 months (*n* = 4) and EPs (*n* = 5). (**C**) TREM2-associated gene signature for patients in the PORTER trial who achieved DC for at least 6 months (*n* = 7) and EPs (*n* = 6). (**D**) Kaplan-Meier PFS curves for patients in the TCGA prostate adenocarcinoma cohort, stratified by high (top 35%) and low (bottom 35%) TREM2-associated gene signature score (*n* = 117 in each group). Box plots show the median and quartiles, and whiskers extend to the largest value no further than 1.5 times the interquartile range. *P* values were calculated using a two-sided Welch’s *t* test or a log-rank test.

We next sought to understand the immune programs contributing to these transcriptional differences between early progressors and patients who achieved disease control. Given that mCRPC harbors a well-reported immunosuppressive TME with an abundance of tumor-associated macrophages (TAMs) (*27*–*31*), we evaluated myeloid-specific gene signatures. Through gene set variation analysis (GSVA) of these transcriptomic data, we derived a TREM2-associated myeloid gene signature consisting of *TREM2*, *APOC1*, *APOE*, *SPP1*, *FABP5*, *CD63*, and *LIPA*. Patients with early progression had a trend toward higher scores for this TREM2-associated gene signature than patients who achieved disease control (*P* = 0.054; Fig. 4B), suggesting that an immunosuppressive TREM2^+^ macrophage-enriched TME may be a key barrier to achieving disease control. These findings align with prior reports implicating TREM2^+^ macrophages in promoting immune evasion and resistance to checkpoint blockade and underscore their potential as candidate resistance biomarkers and therapeutic targets in immunotherapy-refractory tumors (*32*).

To further evaluate the association of this signature with clinical outcomes, we first applied it to an independent cohort of patients with mCRPC from the PORTER trial who received nivolumab-based ICT combinations (*33*). Consistent with our findings, the TREM2-associated myeloid gene signature effectively distinguished patients who progressed within 4 months (*n* = 6) from those who achieved disease control for at least 6 months (*n* = 7, *P* = 0.021; Fig. 4C). Further external validation in the TCGA prostate adenocarcinoma dataset (*34*), which consists of patients with localized disease, demonstrated that patients with the highest signature scores (top 35%) exhibited significantly worse PFS compared to those with the lowest scores (bottom 35%, *n* = 117 in both high and low groups, HR = 2.01, *P* = 0.035; Fig. 4D). These findings suggest that the TREM2-associated myeloid gene signature is associated with disease progression–related outcomes across prostate cancer cohorts.

## DISCUSSION

Despite the limited efficacy of ICTs in mCRPC, combination ipilimumab and nivolumab induced clinical disease control in a subset of patients without evidence of MSI-H or mismatch repair deficient (dMMR) status in the AMADEUS expansion cohort. The observed 6-month DCR of 25.0%, median PFS of 5.4 months, and median OS of 27.9 months are notable given the heavily pretreated nature of the enrolled patients and align with prior trials demonstrating the potential of combination ICTs in mCRPC (*20*–*22*). The ORR and DCR observed in our study were numerically lower than those in the CheckMate 650 trial (*21*), where patients were treated with ipilimumab 3 mg/kg plus nivolumab 1 mg/kg (I3+N1), and the INSPIRE trial (*22*), which tested ipilimumab 1 mg/kg plus nivolumab 3 mg/kg, although direct cross-study comparisons should be approached with caution.

The safety profile of the higher ipilimumab dose cohorts, particularly I5+N1, was consistent with previous studies using various doses (*19*–*21*, *35*), demonstrating the tolerability of high-dose ipilimumab in combination with nivolumab. The frequency and severity of TEAEs were similar across all cohorts and aligned with the established safety profiles of these agents, with no marked increase in AE rates observed in the higher-dose cohorts. Coupled with numerically higher ORR and DCR and the absence of correlation between cumulative ipilimumab dose and disease control, these clinical findings suggest that the lower ipilimumab dose level may offer a more favorable risk-benefit profile.

To better understand the biological mechanisms of anti–CTLA-4 and anti–PD-1 therapy, we performed pharmacodynamic analyses on tissue and blood. Increased median intratumoral CD8 percentages were observed at both low (I1+N360) and high (I5+N1) doses of ipilimumab; however, this increase was not associated with clinical response. Broad immune activation was observed across all three dose cohorts, affecting both CD4^+^ and CD8^+^ T cells and leading to elevated inflammatory mediators, consistent with the increased tumoral CD8 infiltration and known mechanisms of ipilimumab and nivolumab (*17*, *36*, *37*). While most systemic pharmacodynamic changes, including expected increases in ICOS^+^ CD4^+^ T cell subsets (*38*–*40*), were largely consistent across dose cohorts, we observed subtle yet distinct dose-dependent shifts in select proteins and T cell subsets. Despite these differences, the I1+N360 cohort showed a trend toward greater increases in tumoral CD8 percentage. These findings demonstrate that I1+N360 is a biologically active and optimal dose for further clinical investigation in this setting.

In addition to evaluating pharmacodynamic changes, we sought to identify biomarkers to improve mCRPC patient selection for ICTs, which remain limited. The largest study of MSI-H/dMMR and TMB-H prostate cancers (which are present in <5% of patients with prostate cancer) demonstrated clinical responses to ICT in a minority of patients (*41*). Thus, there is a major unmet need for improved biomarkers for patient selection. There was no statistically significant association between clinical outcomes and pretreatment protein levels or T cell subsets. However, patients achieving disease control displayed baseline tumor transcriptional activity in pathways indicative of a more inflamed and immune-active TME, including complement and coagulation cascades, cytokine-cytokine receptor interaction, inflammatory response, and interferon gamma response. This observation is consistent with studies demonstrating that a preexisting immune-activated TME is associated with improved responses to immunotherapy across multiple cancer types, including mCRPC (*12*, *33*, *42*–*45*).

Conversely, patients with early progression exhibited profiles suggestive of distinct barriers to effective immune response. Notably, the TREM2-associated myeloid gene signature (consisting of *TREM2*, *APOC1*, *APOE*, *SPP1*, *FABP5*, *CD63*, and *LIPA*) was enriched in patients with early progression (*P* = 0.054) and significantly associated with poorer outcomes in two independent datasets. However, additional studies will be required to determine whether this signature is prognostic, predictive of response to ICT, or both. *TREM2* plays a key role in myeloid cell function and has been increasingly recognized for its involvement in driving an immunosuppressive TME (*46*, *47*). The inclusion of *APOE* (Apolipoprotein E), a lipid-binding protein highly expressed by TAMs that influences their metabolic state and immunosuppressive functions, further reinforces the myeloid and pro-tumorigenic nature of this signature (*48*–*50*). These results align with emerging evidence from other studies across various cancer types that have identified similar immunosuppressive myeloid cell subsets or gene signatures (often characterized by coexpression of *TREM2*, *APOE*, and *SPP1*), associated with poor prognosis and resistance to immunotherapy (*46*, *48*, *51*). Our group has demonstrated that *SPP1* expression is enriched in patients with mCRPC with the poorest prognosis (*52*). SPP1^+^ TAMs have also been described as mediators of resistance to ICTs in prostate cancer (*30*, *46*). This collective landscape in early progressors, characterized by an immunosuppressive TREM2^+^ myeloid-enriched TME and elevated metabolic pathways, represents a formidable barrier to current immune checkpoint blockade strategies. Together, these findings underscore the rationale for combining ICTs with myeloid-targeted therapies and/or metabolic targeting agents in future trials to overcome such intrinsic resistance mechanisms (*44*, *46*, *53*, *54*).

Several limitations should be considered when interpreting these results. While the overall cohort size was substantial for an expansion study in this patient population, the smaller size of individual dose cohorts, limited matched pre- and on-treatment tumor samples, and the nonrandomized allocation to each cohort limit direct comparative conclusions between regimens. In addition, distinct enrollment timelines and varying nivolumab dose levels across cohorts further restrict definitive cross-cohort comparisons for both efficacy and pharmacodynamics. Although these features may limit the generalizability of these exploratory results, they nonetheless provide a crucial foundation for future investigations of ipilimumab in combination with nivolumab.

In conclusion, the AMADEUS expansion cohort demonstrated that combination ipilimumab plus nivolumab can induce durable disease control in a subset of heavily pretreated patients with mCRPC. This biomarker-rich study provided strong evidence for the I1+N360 regimen as a biologically active and potentially more tolerable dose ratio that may enable rational combinations. This study also identified a TREM2-associated myeloid gene signature that defines a baseline immunosuppressive TME associated with early progression. Currently, novel immunotherapies, such as T cell engagers, are demonstrating promise in prostate cancer; however, challenges remain with respect to durability of responses (*55*). Our findings suggest that ICTs may still have a role in prostate cancer, particularly as part of rational combination strategies with agents such as myeloid-targeted therapies. Targeting immunosuppressive pathways, including the TREM2^+^ macrophage-enriched immune environment, may represent a promising approach to overcome intrinsic resistance and improve outcomes for patients with lethal prostate cancer.

## MATERIALS AND METHODS

### Study design

AMADEUS (NCT03651271) was an open-label, exploratory, multicenter study designed to evaluate nivolumab with or without ipilimumab, with treatment assignment based on the pretreatment percentage of tumoral CD8 cells. The study consisted of two distinct cohorts. The first cohort, comprising patients with various advanced solid tumors, has been previously reported (*19*). Here, we report data from the mCRPC expansion cohort, along with data from patients with mCRPC who were enrolled in the initial solid tumor cohort.

The mCRPC cohort enrolled patients aged ≥18 years with histologically or cytologically confirmed mCRPC who were undergoing androgen deprivation therapy and had progressed on prior second-generation ARPI therapy. Patients in the solid tumor cohort (nivolumab 360 mg plus ipilimumab 1 mg/kg) were not required to have progressed on prior second-generation ARPI therapy. Prior immunotherapy, including anti–PD-(L)1 agents, was permitted if it was not the patient’s most recent line of therapy. Additional key eligibility criteria included a newly obtained core needle or incisional biopsy of a non–bone tumor lesion not previously irradiated, an ECOG performance status of 0 to 1, and measurable disease according to Response Evaluation Criteria in Solid Tumors (RECIST) version 1.1. Key exclusion criteria included a history of grade 3 or higher symptomatic immune-related AEs on prior immunotherapy; untreated brain metastases; active or suspected autoimmune disease; any condition requiring systemic treatment with corticosteroids (>10 mg daily prednisone equivalent) or other immunosuppressive medications within 14 days before starting study intervention; and anticancer chemotherapy, radiotherapy, immunotherapy, or investigational agents within 14 days before starting study intervention.

Patients in the solid tumor cohort received nivolumab 360 mg intravenously (iv) every 3 weeks (Q3W) and ipilimumab 1 mg/kg iv Q3W for the first two doses, followed by ipilimumab 1 mg/kg every 6 weeks (Q6W) for the third and fourth doses (I1+N360 cohort). In the mCRPC cohort, patients received nivolumab 1 mg/kg iv Q3W and either ipilimumab 3 or 5 mg/kg iv Q6W for two doses (I3+N1 and I5+N1 cohorts, respectively). After completing six 3-week cycles in the solid tumor cohort or two 6-week cycles in the mCRPC cohort, patients continued maintenance therapy with nivolumab 480 mg iv every 4 weeks (Q4W) until disease progression or intolerable toxicity. Dose reductions were not permitted but doses could be held for toxicity management. Patients who discontinued ipilimumab due to toxicity were permitted to continue receiving nivolumab. One patient with mCRPC and tumoral CD8 > 15% (CD8 high) was treated with nivolumab monotherapy per protocol design; data from this patient are not reported here.

The mCRPC cohort aimed to enroll approximately 20 patients. The first 10 patients were randomly assigned in a 1:1 ratio to the I3+N1 or I5+N1 cohort. After a safety pause, an additional 10 patients were to be enrolled to I5+N1 if the cohort was deemed tolerable per protocol-defined criteria; otherwise, the remaining patients would enroll to I3+N1. The sample size was not based on formal statistical calculations.

The study protocol and all amendments were approved by the Institutional Review Board of the University of Texas MD Anderson Cancer Center before study initiation and implementation of any protocol changes (Protocol ID: 2017-0446). The study was conducted in accordance with the principles of the Declaration of Helsinki and the International Conference on Harmonization Good Clinical Practice guidelines. All patients provided written informed consent before any study procedures were performed. The trial protocol and statistical analysis plan have been previously published (*19*).

Patients underwent radiographic assessment: I1+N360 cohort within 7 days before day 1 of cycles 4 and 6; I3+N1 and I5+N1 cohorts within 7 days before cycle 2 day 22. All patients continued radiographic assessments every 8 weeks until death, radiographic disease progression, or new therapy initiation. Patients were subsequently followed for survival. AEs were graded according to the National Cancer Institute Common Terminology Criteria for Adverse Events, version 5.0. AE terms were coded using the Medical Dictionary for Regulatory Activities, version 26.0.

For patients in I1+N360, blood sampling for immune biomarkers occurred during cycle 1 days 1 and 8, and day 1 of each subsequent treatment cycle through cycle 4, and at the end of treatment (EOT) visit. For patients in I3+N1 and I5+N1, blood sampling occurred during cycle 1 days 1, 8, and 22; during cycle 2 days 1 and 22; and at EOT. Mandatory core needle tumor biopsies were collected during the screening period from all patients. If medically feasible, on-treatment biopsies were collected during cycles 2 and 6 in I1+N360, and within 3 days of cycle 1 day 22 and cycle 2 day 22 in I3+N1 and I5+N1. Optional biopsies were collected at disease progression in I3+N1 and I5+N1.

### Outcomes

The coprimary endpoints were DCR and change in the percentage of tumoral CD8 cells from baseline. Secondary endpoints were ORR, PFS, OS, and the incidence of AEs. Key exploratory endpoints included the evaluation of tumor and immune biomarkers.

### Translational assays and analysis

Data from CD8 IHC, serum proteomics, mass cytometry time-of-flight (CyTOF) immunophenotyping, WES, and RNA-seq were generated and processed as previously described (*19*), including use of the same reagents and following the same gating strategy. Biomarker analyses were conducted using R version 4.3.2. Outlier samples were identified and excluded based on manual review of the first two principal components in all analyses. Bookend analyses were conducted to evaluate baseline differences between patients with a PR or SD ≥ 6 months (defined as “disease control”) and patients with clinical or radiographic progression within 4 months (before the second tumor assessment, defined as “early progressors”).

RNA-seq analysis: Data were prefiltered to keep genes with ≥10 counts in at least three samples. Differential expression analysis was performed using DESeq2 (version 1.42.0) (*56*), using a significance threshold of Benjamini-Hochberg adjusted *P* value <0.05. GSEA was performed using fgsea (version 1.28.0) on the Hallmark and KEGG gene sets provided by the msigdbr package (version 7.5.1), with significant pathways having a Benjamini-Hochberg adjusted *P* value <0.01.

Gene signature identification and validation: GSVA (*57*) was applied to the pretreatment tumor RNA-seq data to evaluate the enrichment of myeloid-specific gene signature programs. To validate the identified signature, its score was calculated in independent cohorts using GSVA. In the PORTER dataset (NCT03835533) (*33*), signature scores were compared between patients with disease control ≥6 months and early progressors (clinical or radiographic progression within 4 months) using a Welch’s two-sided *t* test. For the TCGA prostate adenocarcinoma dataset (*34*), patients were stratified into “high” (top 35%) and “low” (bottom 35%) signature score groups, and PFS was compared between these groups using a log-rank test.

Serum proteomics and CyTOF immunophenotyping analysis: Protein levels and CyTOF data were batch adjusted by treatment cohort using ComBat from the sva package (version 3.50.0). For baseline versus on-treatment differences, two-sided *t* tests were performed with a Bonferroni-adjusted *P* value threshold of <0.05 and mean difference >0.5 for proteins or absolute log2 fold change >0.5 for cell populations. Comparisons of baseline versus on-treatment effects between I1+N360 and I5+N1 cohorts were analyzed using two-sided paired *t* tests of absolute differences (C1D8 − C1D1) with an unadjusted *P* value threshold of <0.05 and, for proteins only, mean difference > 0.25.

### Statistical analysis

Efficacy and safety analyses were conducted on all patients who received at least one dose of study intervention. Analyses of changes in CD8 percentage were conducted on all patients with at least one on-treatment biopsy with sufficient CD8 results. Patients were grouped according to the treatment group assigned at enrollment. This study was not intended or powered for statistical comparison between groups and no adjustment for multiple comparisons was performed for the clinical endpoints.

DCR (referred to as clinical benefit rate in the protocol and statistical analysis plan) was defined as the proportion of patients with best overall response of complete response (CR) or PR, or SD lasting at least 6 months per investigator-assessed RECIST version 1.1. Confirmation of response by a repeat tumor assessment was required for a best overall response of CR or PR. CIs for DCR were calculated using the Clopper-Pearson method.

ORR was defined as the proportion of patients with a best response of CR or PR; PFS was defined as the time from treatment initiation until radiographic disease progression or death (whichever occurred first); and OS was defined as the time from treatment initiation until death due to any cause. CIs for ORR were calculated using the Clopper-Pearson method. The Kaplan-Meier method was used to estimate PFS, OS, and the corresponding CIs. Statistical analyses of clinical data were performed using SAS version 9.4 and R version 4.3.2.
