# Supplementary material for: Immune biomarkers of response and resistance to ipilimumab and nivolumab in metastatic castration-resistant prostate cancer
Source: Sci Adv. 2026 Oct 14;12(42):eaee8730. doi: 10.1126/sciadv.aee8730 (PMC13666023; doi:10.1126/sciadv.aee8730)
Supplement: Supplementary file 1 [file sciadv.aee8730_sm.pdf]

Science Advances
AAAS

Supplementary Materials for

Immune biomarkers of response and resistance to ipilimumab and nivolumab in metastatic castration-resistant prostate cancer

Apostolia M. Tsimberidou *et al.*

Corresponding author: Padmanee Sharma, padsharma@mdanderson.org

Sci. Adv. **12**, eaee8730 (2026)
DOI: 10.1126/sciadv.aee8730

The PDF file includes:

Figs. S1 to S4
Tables S1 to S5
Legends for data files S1 and S2

Other Supplementary Material for this manuscript includes the following:

Data files S1 and S2

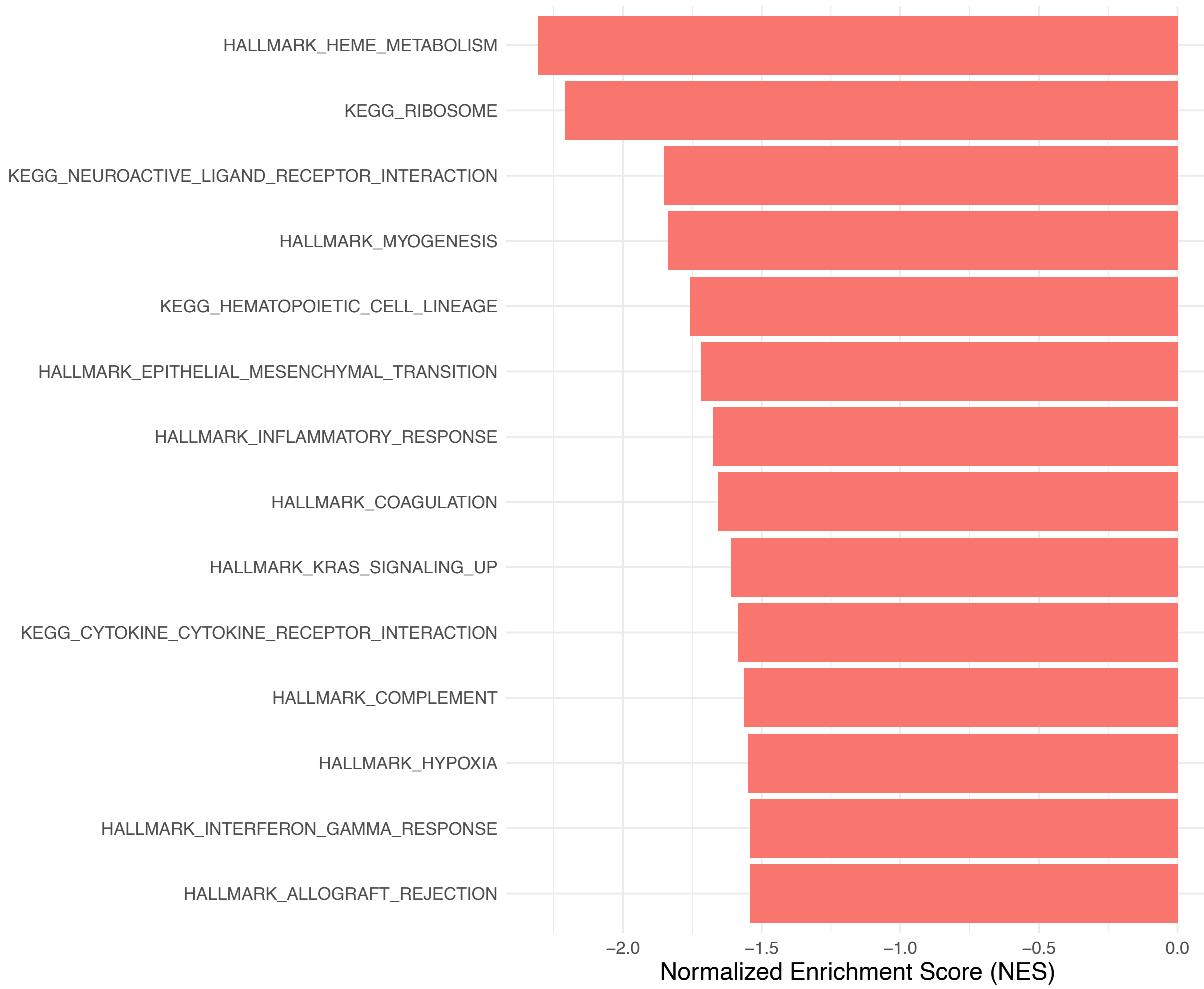

Supplementary Figure S1: Gene Set Enrichment Analysis (GSEA) of RNA-seq data from matched pre- and on-treatment tumor biopsies. GSEA was performed on pooled data from 6 patients across the three treatment cohorts (n = 1, 1, and 4 for I1+N360, I3+N1, and I5+N1, respectively). Pathways significantly affected by treatment, with a Benjamini-Hochberg adjusted p-value < 0.01, are displayed. Red bars indicate pathways downregulated following ipilimumab and nivolumab treatment.

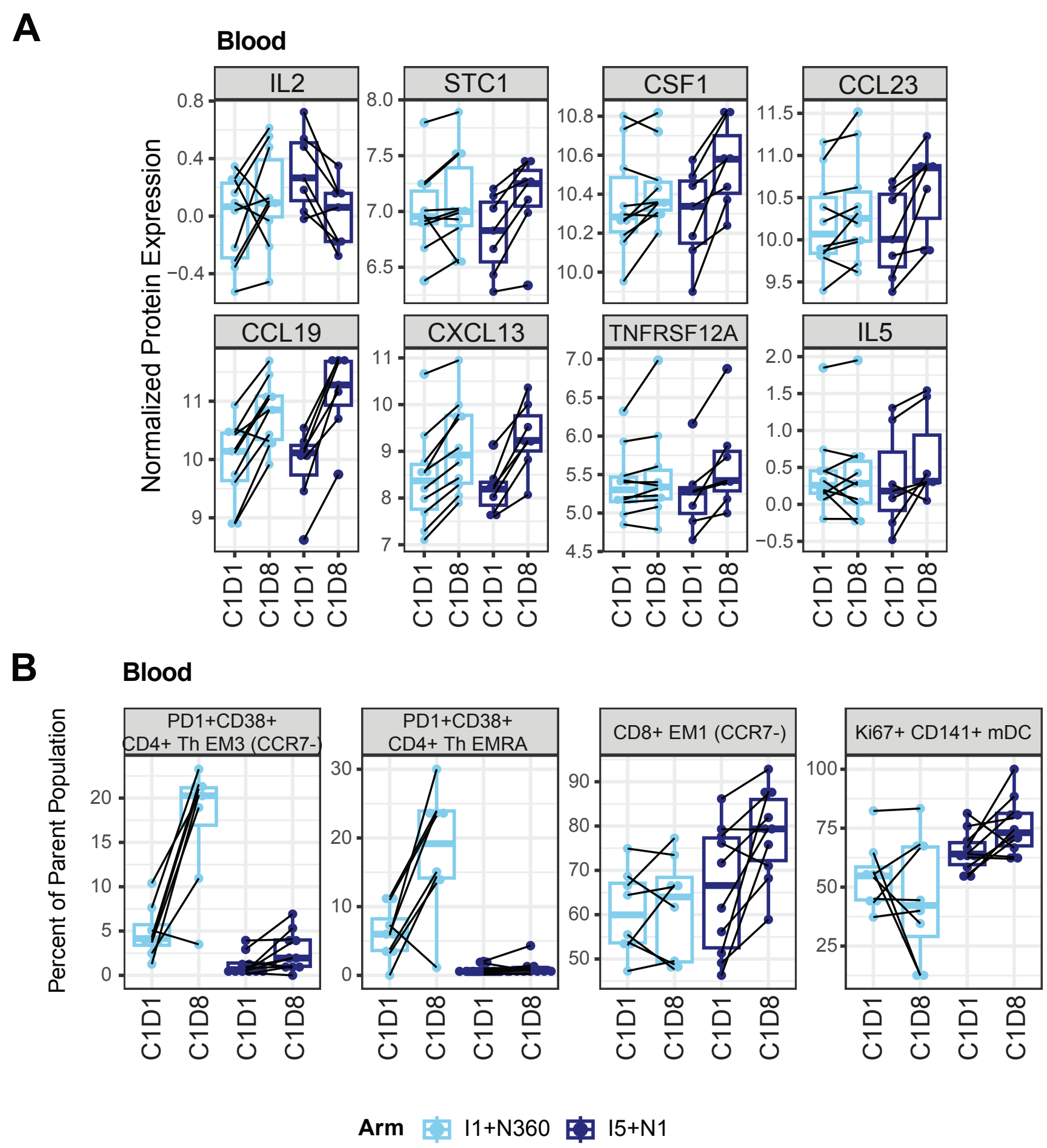

Supplementary Figure S2: Changes in blood-based candidate biomarkers in response to ipilimumab and nivolumab. Boxplots showing (**A**) serum proteins and (**B**) cell populations with differences in the magnitude of pharmacodynamic effects at Cycle 1 Day 8 (C1D8) between the I1+N360 and I5+N1 treatment cohorts. Box plots show the median and quartiles, and whiskers extend to the largest value no further than 1.5 times the interquartile range. C1D1 = Cycle 1 Day 1; EM = effector memory; EMRA = terminally differentiated effector memory; mDC = myeloid dendritic cell; Th = T helper cell.

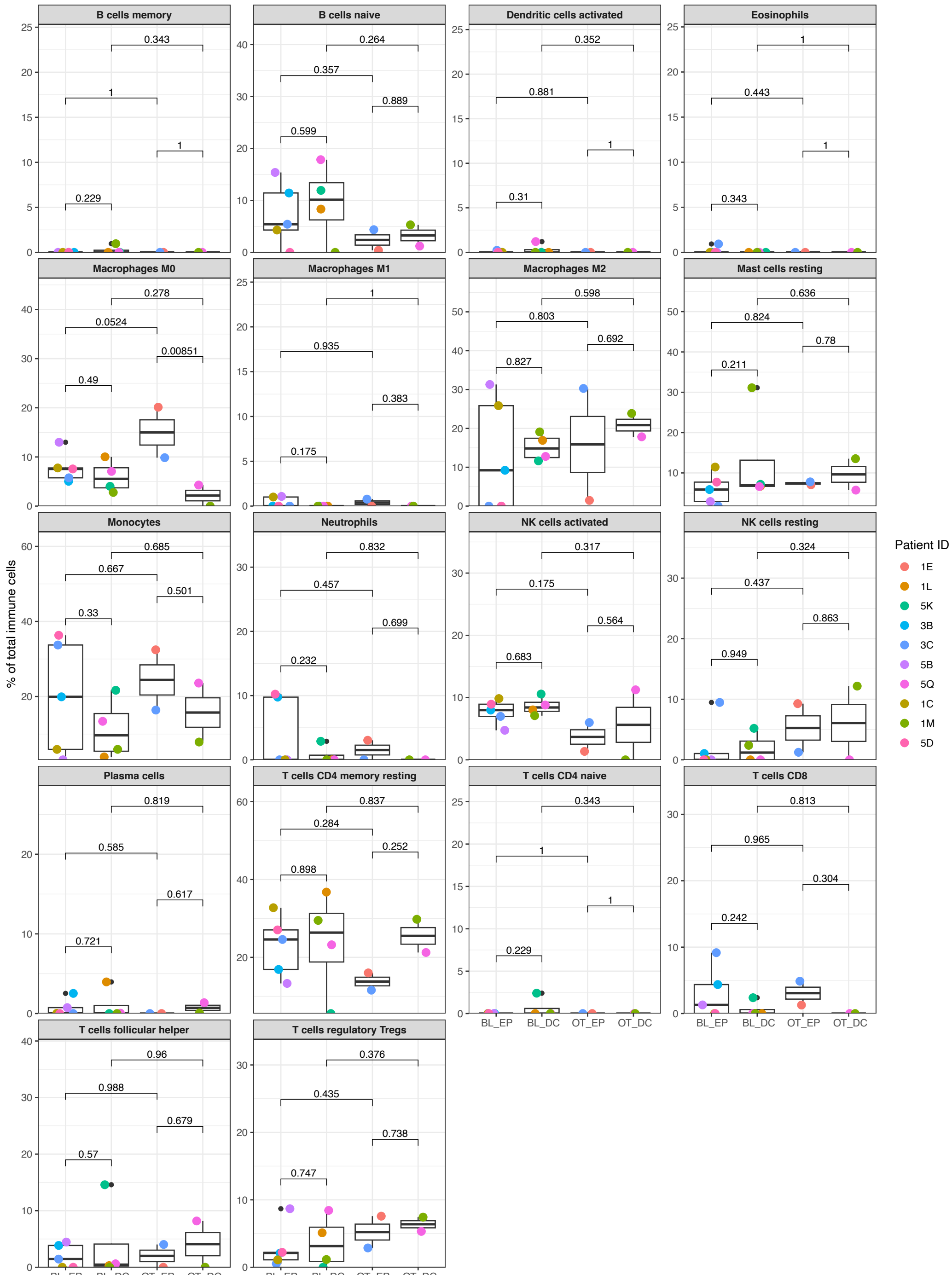

Supplementary Figure S3: CIBERSORT analysis of pre- and on-treatment tumor biopsies. Relative immune cell composition was estimated using the CIBERSORT algorithm applied to bulk RNA-seq data from baseline (BL) and on-treatment (OT) tumor samples in patients with early progression (EP) or disease control (DC). No statistically significant differences in immune cell proportions were observed between groups (t test). Estimates are based on the LM22 leukocyte signature matrix, which captures major immune populations without fine subset resolution.

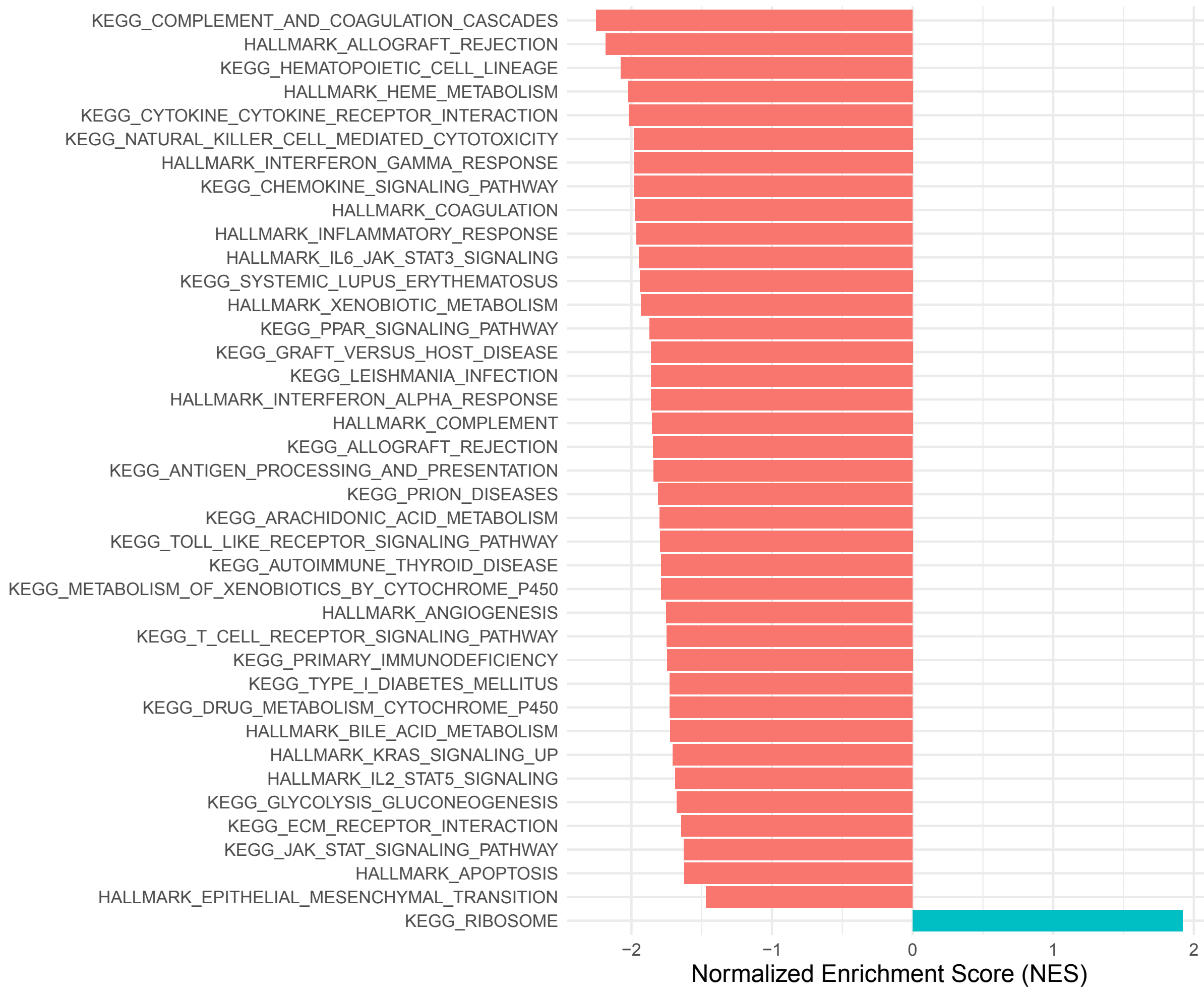

Supplementary Figure S4: Gene Set Enrichment Analysis (GSEA) of baseline tumor RNA-seq data between patients achieving disease control and early progressors. Pathways with Benjamini-Hochberg adjusted p-value < 0.01 are displayed: red bars indicate pathways upregulated in patients with early progression and blue bars indicate pathways upregulated in patients with disease control.

Supplementary Table S1: Prior systemic cancer therapies.

Drug Class	Agent(s)	I1+N360 (N = 12)	I3+N1 (N = 5)	I5+N1 (N = 15)	Total (N = 32)
2nd generation androgen-receptor pathway inhibitor (ARPI)	Abiraterone, Apalutamide, Darolutamide, Enzalutamide, TRC253	11 (92)	5 (100)	15 (100)	31 (97)
Chemotherapy	Docetaxel, Cabazitaxel, Cabazitaxel + Carboplatin, Carboplatin, Imifoplatin, Mitoxantrone	8 (67)	4 (80)	10 (67)	22 (69)
Radiopharmaceutical therapy	Radium (Ra 223) dichloride, Lutetium (177Lu) vipivotide tetraxetan, Actinium-225-PSMA-617	2 (17)	2 (40)	1 (7)	5 (16)
Vaccine	Rilimogene galvacirepvec + Rilimogene glafolivec, Sipuleucel-T	3 (25)	2 (40)	9 (60)	14 (44)
Anti-CTLA-4 therapy	Tremelimumab	1 (8)	0	0	1 (3)
Anti-PD-(L)1 therapy	Cetrelimab, Durvalumab, Pembrolizumab, Zimberelimab	1 (8)	1 (20)	2 (13)	4 (13)
Poly ADP ribose polymerase (PARP) inhibitor	Niraparib, Olaparib	0	1 (20)	2 (13)	3 (9)
Ataxia telangiectasia and Rad3-related (ATR) kinase inhibitor	RP-3500	0	1 (20)	0	1 (3)
Bromodomain and extra-terminal domain (BET) inhibitor	ZEN-3694	1 (8)	0	0	1 (3)
Phosphatidylinositol-3-Kinase (PI3K) inhibitor	GSK2636771	0	1 (20)	0	1 (3)

Supplementary Table S2: Exposure to ipilimumab and nivolumab.

	**I1+N360 (N = 12)**	**I3+N1 (N = 5)**	**I5+N1 (N = 15)**
Ipilimumab			
Number of doses received, n (%)			
1	2 (17)	2 (40)	7 (47)
2	5 (42)	3 (60)	8 (53)
3	2 (17)	--	--
4	3 (25)	--	--
Cumulative dose (mg), median (range)	228.0 (83.0 – 485.2)	480.0 (275.0 – 511.6)	690.0 (256.5 – 1,074.0)
Nivolumab			
Number of doses received, median (range)	3 (1 – 21)	3 (1 – 11)	3 (1 – 10)
Cumulative dose (mg), median (range)	1,080.0 (360.0 – 9,240.0)	243.0 (91.0 – 3,692.0)	304.0 (51.3 – 3,231.6)

Supplementary Table S3: Demographic and baseline disease characteristics by disease control status.

	Achieved Disease Control (N = 8)	No Disease Control (N = 24)	P-value
Age, median (range), years	76.0 (63 – 80)	67.5 (50 – 84)	0.067
Race, n (%)			0.296
Black or African American	0	5 (21)	
White	8 (100)	18 (75)	
Other	0	1 (4)	
ECOG performance status at Screening, n (%)			0.176
0	4 (50)	5 (21)	
1	4 (50)	19 (79)	
Prior lines of cancer therapy, median (range)	3.5 (2 – 9)	5.0 (3 – 10)	0.113
Received prior anti-PD-(L)-1 therapy, n (%)	1 (13)	3 (13)	1.000
Received prior anti-CTLA-4 therapy, n (%)	0	1 (4)	1.000
PSA (ng/mL) at Baseline, median (range)	25.2 (1.9 – 761.5)	68.6 (0.0 – 4,271.0)	0.372
Tumoral CD8 cells (%) at Screening, median (range)	3.2 (1.0 – 9.8)	3.4 (0.0 – 14.1)	0.728
Alkaline phosphatase, n (%)			0.298
< 1.5 x ULN	8 (100)	17 (71)	
≥ 1.5 x ULN	0	6 (25)	
Not reported	0	1 (4)	
Hemoglobin, n (%)			0.035
< 11 g/dL	0	10 (42)	
≥ 11 g/dL	8 (100)	14 (58)	
Lactate dehydrogenase, n (%)			0.027
< 200 U/L	5 (63)	3 (13)	
≥ 200 U/L	3 (38)	16 (67)	
Not reported	0	5 (21)	

Abbreviations: ECOG = Eastern Cooperative Oncology Group, ULN = upper limit of normal. P-values were calculated using a two-sided Fisher's exact test for categorical variables and a two-sided Mann-Whitney test for continuous variables.

Supplementary Table S4: Treatment-emergent adverse events (TEAEs) observed in ≥ 10% of patients.

MedDRA v26.0 Term, n (%)	I1+N360 (N = 12)	I3+N1 (N = 5)	I5+N1 (N = 15)	Total (N = 32)
Fatigue	6 (50)	3 (60)	4 (27)	13 (41)
Rash	5 (42)	2 (40)	4 (27)	11 (34)
Diarrhea	4 (33)	2 (40)	3 (20)	9 (28)
Dyspnea	4 (33)	1 (20)	3 (20)	8 (25)
Nausea	2 (17)	1 (20)	5 (33)	8 (25)
Anemia	4 (33)	2 (40)	1 (7)	7 (22)
Constipation	2 (17)	1 (20)	4 (27)	7 (22)
Vomiting	2 (17)	1 (20)	4 (27)	7 (22)
Alanine aminotransferase increased	2 (17)	1 (20)	3 (20)	6 (19)
Aspartate aminotransferase increased	2 (17)	1 (20)	3 (20)	6 (19)
Cough	3 (25)	0	3 (20)	6 (19)
Dehydration	4 (33)	1 (20)	1 (7)	6 (19)
Hypokalemia	1 (8)	2 (40)	3 (20)	6 (19)
Decreased appetite	1 (8)	2 (40)	2 (13)	5 (16)
Hyponatremia	1 (8)	2 (40)	2 (13)	5 (16)
Weight decreased	1 (8)	0	4 (27)	5 (16)
Back pain	1 (8)	0	3 (20)	4 (13)
Blood creatinine increased	2 (17)	1 (20)	1 (7)	4 (13)
Hypocalcemia	1 (8)	1 (20)	2 (13)	4 (13)
Hypothyroidism	1 (8)	0	3 (20)	4 (13)
Muscular weakness	3 (25)	0	1 (7)	4 (13)

Supplementary Table S5: Treatment discontinuations due to an adverse event.

Cohort	Patient ID	Adverse Event Term	Toxicity Grade	Study Drug(s) Assessed as Related per Investigator	# Ipilimumab Doses Received	# Nivolumab Doses Received
I1+N360	1L	Alanine aminotransferase increased	Grade 3	Ipilimumab, Nivolumab	1	1
	1F	Thrombocytopenia (owing to bone marrow involvement)	Grade 3	Unrelated	2	2
	1J	Dyspnea	Grade 2	Unrelated	3	4
	1B	Rhabdomyolysis	Grade 3	Ipilimumab, Nivolumab	2	2
I5+N1	5H	Immune-mediated colitis	Grade 3	Ipilimumab, Nivolumab	1	2
	5J	Immune-mediated colitis	Grade 3	Ipilimumab, Nivolumab	2	3
	5L	Immune-mediated colitis	Grade 3	Ipilimumab, Nivolumab	1	1
	5F	Immune-mediated hepatitis	Grade 4	Ipilimumab, Nivolumab	1	3
	5K	Polymyalgia rheumatica	Grade 2	Ipilimumab, Nivolumab	2	9

Data file S1: Listing of disease characteristics and outcomes for patients with disease control.

Data file S2: Differentially expressed genes in pre-treatment tumors between patients who achieved disease control (*n* = 4) and those with early progression (*n* = 5). Differentially expressed genes were identified using DESeq2. Positive log fold change values indicate genes with higher expression in patients who achieved disease control. Adjusted p-values were calculated using the Benjamini-Hochberg method.
